# TNF-α regulates the composition of the basal lamina and cell-matrix adhesions in gingival epithelial cells

**DOI:** 10.1080/19336918.2022.2029237

**Published:** 2022-02-09

**Authors:** Masaru Mezawa, Yuto Tsuruya, Arisa Yamaguchi, Mizuho Yamazaki-Takai, Tetsuro Kono, Hiroyuki Okada, Christopher A. McCulloch, Yorimasa Ogata

**Affiliations:** a Department of Periodontology, Nihon University School of Dentistry at Matsudo, Matsudo, Japan; b Research Institute of Oral Science, Nihon University School of Dentistry at Matsudo, Japan; c Department of Histology, Nihon University School of Dentistry at Matsudo, Matsudo, Japan; d Faculty of Dentistry, University of Toronto, Toronto, Canada

**Keywords:** TNF-α, gingival epithelial, cell adhesion, cell migration, laminin 5, basal lamina, extracellular matrix

## Abstract

Laminin 5, type 4 collagen, and α6β4 integrin contribute to the formation of hemidesmosomes in the epithelia of periodontal tissues, which is critical for the development and maintenance of the dentogingival junction. As it is not known whether TNF-α alters the composition of the epithelial pericellular matrix, human gingival epithelial cells were cultured in the presence or absence of TNF-α. Treatment with TNF-α accelerated epithelial cell migration and closure of in vitro wounds. These data indicate unexpectedly, that TNF-α promotes the formation of the pericellular matrix around epithelial cells and enhances adhesion of epithelial cells to the underlying matrix, properties which are important for cell migration and the integrity of the dentogingival junction.

## Introduction

Tumor necrosis factor-alpha (TNF-α) is a prominent, proinflammatory mediator released from monocytes and macrophages [1] that is broadly proinflammatory and immunomodulatory for discrete cell populations. These effects arise in part from enhanced prostaglandin synthesis and are associated with tumor formation in a variety of cancers, increased protease expression, osteoclast activation and bone resorption. TNF-α upregulates the production of the interstitial collagenase, matrix metalloproteinase-1 (MMP-1), prostaglandin E2 (PGE2), myriad cytokines and chemokines, cell adhesion molecules, and molecules that promote bone resorption [2–4]. MMPs are required for the degradation of the extracellular matrix (ECM), a process that is crucial for tumor growth, invasion and metastasis [5]. Notably, TNF-α is also upregulated in periodontitis [6,7], a high prevalence inflammatory disease. It is not known in detail how TNF-α affects the structure and protein composition of the epithelial components of the dentogingival junction.

In the dentogingival junction, the junctional epithelium adheres to the tooth surface and exhibits two distinct basal laminae. The external lamina is continuous with the basal lamina of the sulcular epithelium and attaches the junctional epithelium to the underlying lamina propria of the connective tissue. The internal basal lamina attaches the junctional epithelium to the tooth surface [8,9] through hemidesmosomes, which are comprised of laminin 5 and integrin α6β4 [10–12]. Based on immunohistochemistry and *in situ* hybridization, laminin 5 is located in the internal basal lamina of the junctional epithelium [13]. Laminin 5 is found only in the internal basal lamina, which lacks type IV collagen. Laminin 5 and type IV collagen are both present in the ECM of the lamina propria of the gingival connective tissue [14].

Plectin can interact with itself, with intermediate filament proteins such as keratins, and with multiple domains in the β4 integrin tail. Plectin contributes to the clustering of the α6β4 integrin at the basal surface of the cell, a critical step in the formation of hemidesmosomes [15–17]. Cytokeratins interact with the nuclear membrane and also with desmosomes and hemidesmosomes at the plasma membrane; these interactions enhance the structural integrity of epithelial cells [18]. Protein interactions with membrane proteins are often mediated by members of the plakin family including desmoplakin, plectin and certain integrins. Further, in the basal lamina, the odontogenic ameloblast-associated protein (ODAM) is implicated in diverse activities such as ameloblast differentiation, junctional epithelial attachment to teeth [19] enamel maturation, and tumor growth [20,21].

The relationship between oral squamous epithelial cancers and the behavior of epithelia in periodontal diseases has been discussed earlier [22–24]. Notably, neoplastic diseases may occur as primary lesions of periodontal tissues or as secondary metastatic neoplasms. The clinical features of oral squamous cell carcinoma can resemble the migration of pocket epithelial cells in to the lamina propria of the gingiva as is seen in localized periodontitis or acute periodontal infection in which there is gingival erythema, swelling, increased probing pocket depths, and radiographic evidence of bone loss [25]. In view of this relationship, we used human gingival squamous carcinoma cells (Ca9-22) as a cellular model to investigate whether TNF-α regulates the structure and function of the pericellular matrix and MMP expression. We found that TNF-α strongly influences protein expression and the composition of the basal lamina, suggesting that TNF-α may directly impact the structure and metabolism of pericellular proteins, which in turn regulate the adhesion of junctional epithelial cells to the enamel.

## Materials and methods

### Reagents

Rabbit polyclonal antibodies (laminin 5, type IV collagen, type I collagen and p115-rhoGEF), rabbit monoclonal antibodies (the 67 kDa laminin receptor, Tak1 and TIMP1), mouse monoclonal antibodies (β4 integrin, MMP-9 and cytokeratin 19), phalloidin-iFluor 488 reagent, goat anti-rabbit Alexa 488, and goat anti-mouse Alexa 647 were obtained from Abcam (Tokyo, Japan). Rabbit polyclonal antibodies to plectin were purchased from Novus (Littleton, CO). Mouse monoclonal antibodies to β-actin were purchased from Cell Signaling Technology. Rabbit polyclonal antibodies to odontogenic ameloblast-associated protein (ODAM) were purchased from Proteintech (Rosemont, IL). Rat monoclonal antibodies to the α6 integrin (CD49f; GoH3) were purchased from R & D Systems (Minneapolis, MN). Human recombinant tumor necrosis factor-α (TNF-α), interleukin-1β (IL-1β) and alpha-minimum essential medium (α-MEM) were purchased from Wako (Tokyo, Japan). Fetal calf serum (FCS), penicillin and streptomycin, TrypLE Express, and TRIzol Reagent were purchased from Invitrogen (Carlsbad, CA). The PrimeScript RT reagent kit and SYBR Premix Ex Taq II were purchased from Takara Bio (Tokyo, Japan). Bovine serum albumin (BSA), complete protease inhibitor cocktail, and phenylmethylsulfonyl fluoride were purchased from Sigma Aldrich Japan (Tokyo, Japan). Anti-mouse rabbit IgG (whole molecule) conjugated to peroxidase antibody, anti-rabbit goat IgG (whole molecule) conjugated to peroxidase, and ECL Prime Western blotting Detection Reagents were purchased from GE Healthcare (Buckinghamshire, UK). Fibronectin was from bovine plasma (Sigma-Aldrich). All chemicals used were of analytical grade.

### Cell culture

Human gingival squamous carcinoma epithelial Ca9-22 cells purchased from the RIKEN BRC Cell Bank (Tsukuba, Japan). Ca9-22 cells were cultured in αMEM containing 10% fetal calf serum until 70–80% confluent in 5% CO2 and 95% air at 37°C. Ca9-22 cells were stimulated in α-MEM containing 1% fetal calf serum with TNF-α (10 ng/ml) or IL-1β (1 ng/mL) for 6 h or 24 h.

### Real-time PCR

Total RNA was isolated using TRIzol Reagent according to the manufacturer’s protocol. Total RNA (1 mg) was used as a template for cDNA, which was prepared with the PrimeScript RT reagent kit. With the use of SYBR Premix Ex Taq II in a TP800 Thermal Cycler Dice Real-Time System (Takara Bio), we performed quantitative real-time PCR with specific primer sets (Table 1). The amplification reactions were performed in 25 mL final volumes and contained ×2 SYBR Premix EX Taq (12.5 mL), forward and reverse primers (0.2 mL), and 70 ng cDNA (7 mL) for TNF-α, MMP-2, MMP-9, TIMP-1, β4 integrin, cadherin1, laminin β3, laminin γ2, laminin α3, type IV α1 collagen chain and type I α1 collagen chain; 50 ng cDNA (5 mL) was used for analyzing GAPDH. To reduce variability between replicates, PCR pre-mixes containing all reagents except for cDNA, were prepared and aliquoted into 0.2 mL PCR tubes (Nippon Genetics). The thermal cycling conditions were: 10s at 95°C, 40 cycles of 5 s at 95°C and 30s at 60°C. Post-PCR melting curves confirmed the specificity of single-target amplification and the resultant mRNA expression. TNF-α, MMP-2, MMP-9, TIMP-1, β4 integrin, cadherin1, laminin β3, laminin γ2, laminin α3, type IV α1 collagen and type I α1 collagen and normalized to GAPDH data were measured in triplicate for each experimental condition and on 3 different days.

**Table 1.** Human primers used for real-time PCR

TNF-α
forward 5′ -GTGACAAGCCTGTAGCCCATGTT-3’
reverse 5′ -TTATCTCTCAGCTCCACGCCATT-3’
MMP-2
forward 5′ -GATACCCCTTTGACGGTAAGGA-3’
reverse 5′ - CCTTCTCCCAAGGTCCATAGC-3’
MMP-9
forward 5′ -ACGCACGACGTCTTCCAGTA-3’
reverse 5′ -CCACCTGGTTCAACTCACTCC-3’
TIMP-1 (tissue inhibitor of metalloproteinase-1)
forward 5′ -AAGACCTACACTGTTGGCTGTGAG-3′;
reverse 5′ -GTCCGTCCACAAGCAATGAG-3′;
β4 Integrin
forward 5′ -CTCCACCGAGTCAGCCTTC-3′
reverse 5′ -CGGGTAGTCCTGTGTCCTGTA-3′
Cadherin 1
forward 5′ -AAGTGCTGCAGCCAAAGACAGA-3′
reverse 5′ -AAATTGCCAGGCTCAATGACAAG-3′
Laminin β3-chain
forward 5′ - CCAAGCCTGAGACCTACTGC-3′;
reverse 5′ -GAATCTCCTGTCCAGGTCCA-′3
Laminin γ2-chain
forward, 5′ -GACAAACTGGTAATGGATTCCGC-3′;
reverse, 5′ -TTCTCTGTGCCGGTAAAAGCC-3′
Laminin α3-chain
forward, 5′ -TGCTAACAGTATCCGGGATTCT-3′;
reverse, 5′ -TCTTGGTTCAAGCCATTTGCC-3′
Type IV collagen α1-chain
forward, 5′ -ACCTGGTCAAACTGGTCCTG-3′;
reverse, 5′ -GTGTCCCCTAATGCCTTTGA-3′;
Type I collagen α1-chain
forward, 5′ -GCTTGGTCCACTTGCTTGAAGA-3′;
reverse, 5′ -GAGCATTGCCTTTGATTGCTG-3′
GAPDH (glyceraldehyde-3-phosphate dehydrogenase)
forward, 5′ -GCACCGTCAAGGCTGAGAAC-3′;
reverse, 5′ -ATGGTGGTGAGACGCCAGT-3′

### Western blotting

Ca9-22 cells were lysed and equal amounts of protein were loaded and separated on 8% SDS-PAGE gels, and transferred to Hybond 0.2-μm polyvinylidene fluoride membranes. The membranes were blocked and probed with specific primary antibodies at 4°C overnight, followed by secondary antibody incubation for 1 h at room temperature. Membranes were incubated with anti-TAK1 (ab109526; Abcam, Cambridge, UK), anti-MMP-9 (ab119906; Abcam), anti-β4 integrin (ab29042; Abcam), anti-cytokeratin 19 (ab7755; Abcam), anti-p115-rhoGEF (ab220892; Abcam), anti-laminin 5 (ab14509; Abcam), anti-67 kDa laminin receptor (ab133645; Abcam), anti-type IV collagen (ab6586; Abcam), and anti-β-actin (#12,262; Cell Signaling Technology, MA) antibodies for 2 h. Anti-rabbit and anti-mouse IgGs conjugated with horseradish peroxidase were used as the secondary antibodies. Immunoreactivity was detected by ECL. The ChemiDoc MP Imaging System (Bio-Rad, Hercules, CA) was used for detection and analysis of immunoblots.

### Cell migration assay

Cell migration was determined with Radius™ 24-well plates (from Cell Biolabs, San Diego, CA). Plates were seeded with 5 × 10^3^ Ca9-22 cells/ml per well. Cells were cultured in α-MEM containing 10% FCS on plates coated with fibronectin. Cells were cultivated to form monolayers before circular gaps were generated by removing gel inserts that had been positioned prior to cell culture. Cells in α-MEM-containing 1% FCS were treated with IL-1β (1 ng/mL) or TNF-α (10 ng/mL) for 6 h or 24 h. After treatments, cells were washed with PBS and fixed in 4% paraformaldehyde for 10 min. After three washes with PBS, cells were stained with DAP1 for 15 min and the width of the gaps in the cultures was measured at the same magnification (×4) using a fluorescence microscope (BZ-X810; Keyence, Japan). To block integrin function, neutralizing antibodies to integrin α6 were incubated with Ca9-22 cells cultured in αMEM with 10% FCS in 35-mm culture dishes and grown to confluence. The medium was changed to α-MEM containing 1% FCS and cell layers were scratched with a cell scraper (1 mm wide; Corning) in the center of the dishes, which were washed twice with α-MEM containing 1% FCS to remove detached cells. Cells were stimulated with TNF-α (10 ng/mL) and treated with integrin‐neutralizing antibodies (10 μg/ml) for 24 h to observe the wound healing area.

### Immunofluorescence analysis

Ca9-22 cell suspensions were prepared by trypsinization and were incubated with 0.5 mg/ml bacterial collagenase for 30 min at 37°C with agitation to remove the pericellular glycocalyx. Chamber slides (8 wells) were seeded with 1 × 10^4^ cells/ml per chamber, and the cells were cultured in α-MEM with 10% FCS for 12 h. Countess Cell Counting Chamber Slides (Invitrogen) were used for cell counting. Cells were washed with PBS (137 mM NaCl, 2.7 mM KCl, 8 mM Na_2_HPO_4_, and 1.5 mM KH_2_PO_4_; pH7.4). Chamber slides (8-well; Corning) were coated with 10 μg/mL of fibronectin at 37°C for 60 min. The medium was changed to α-MEM with 1% FCS for 6 h, and the cells were then treated with 10 ng/mL TNF-α for 6 h or 24 h. After treatment, cells were fixed in 4% paraformaldehyde for 10 min. After three washes with PBS, cells were permeabilized with 0.1% Triton X-100 for 5 min. In experiments to quantify the synthesis of pericellular basal lamina proteins, permeabilization with Triton X-100 was not performed. Cells were blocked in 2.5% goat serum in 4% BSA for 30 min at room temperature. After one wash with PBS, the primary antibodies (rabbit polyclonal anti-laminin 5, anti-type IV collagen, anti-ODAM and anti-plectin antibody, and mouse monoclonal anti-integrin β4) were used at a 1:200 concentration for 2 h at 37°C. After three washes with PBS, the secondary antibodies (Alexa Fluor 488 and Alexa Fluor 647 goat anti-rabbit IgG) were used at a 1:200 concentration for 1 h at room temperature. After three washes with PBS, coverslips were mounted with AntiFade Poly/Mount with DAPI(Polysciences, Warrington, PA, USA). Fluorescence images were viewed under a Zeiss LSM 510 (Oberk-ochen, Germany) confocal microscope.

### Statistical analysis

For all experiments, separate assays were repeated at least three times on different days. For quantitative data, means ± SEM were computed. Comparisons of multiple samples were analyzed with ANOVA. Statistical significance was set at p < 0.05.

## Results

### TNF-α regulates extracellular matrix digestion

**Figure 1.**
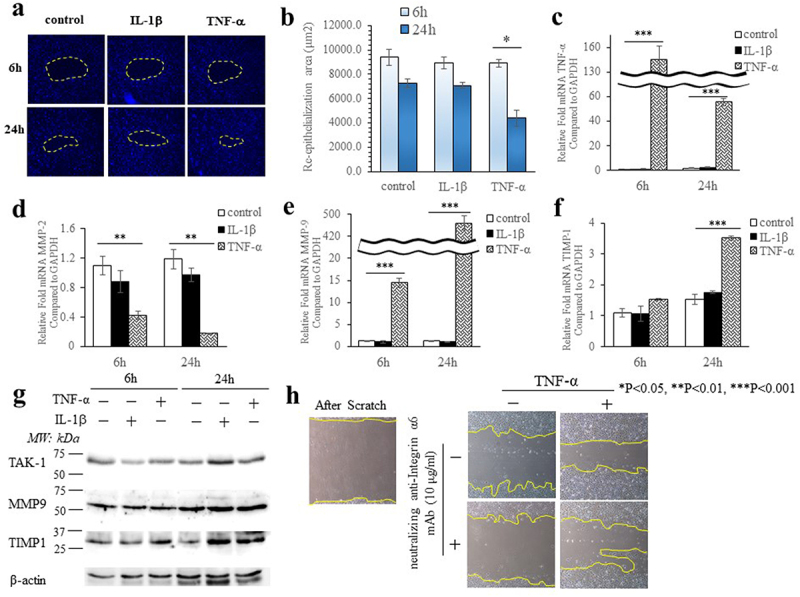
TNF-α regulates extracellular matrix digestion pathways. (a) Wound healing analyses on re-epithelialization. Re-epithelialization in Ca9-22 cells was analyzed on Radius™ 24- well plate (fibronectin-coated). Observation with fluorescence microscope was performed 6 h and 24 h after tread IL-1β and TNF-α. Three different cellular stains were used a nuclear DAPI stain. (b) Mean wound healing area (re-epithelialization) in IL-1β and TNF-α-treated cell is shown in panel. The data represent the results of three experiments. Effects of IL-1β (1 ng/ml) and TNF-α (10 ng/ml) on mRNA and protein levels of extracellular matrix remodeling factors in Ca9-22 cells. Quantification of mRNA expression of (c) TNF-α, (d) MMP-2, (e) MMP-9 and (f) TIMP-1 in Ca9-22 cells that were treated IL-1β and TNF-α for 6 or 24 h before RNA extraction. Data are means SEM ± from 3 separate experiments. (g) Immunoblot analysis for TAK1, MMP-9 and TIMP1 of lysates prepared from Ca9-22 cells. β-actin was used as a loading control. The data are representative of 3 separate experiments. * p < 0.05, ** p < 0.01, *** p < 0.001. (h) Effects of α6 integrin neutralizing antibodies (GoH3) on cell migration. Migration assays were performed for 24 h using Ca9-22 cells stimulated with either or both TNF-α (10 ng/ml) and integrin‐neutralizing antibodies (10 μg/ml). Observation by microscopy showed that TNF-α-treated wounded cells wounds closed rapidly after scratching. The panels show cells immediately after scraping (left panel) and 24 h later (right panels).

We examined the effect of IL-1β (1 ng/ml) and TNF-α (10 ng/ml) on the migration of cultured Ca9-22 cells and their ability to close in vitro gaps after 6 h or 24 h of cytokine treatments (Figure 1(a)). The mean percentage of cell closure of the gaps was reduced more after treatment with TNF-α (24 h) than with TNF-α for 6 h (p < 0.05, Figure 1(b)). There was no difference in the percentage of gap closure between controls or IL-1β treated-cells. We examined TNF-α, MMP-2, MMP-9, TIMP-1 and TAK1 expression to assess ECM degradation in Ca9-22 cells (Figure 1(b)). Ca9-22 cells were treated with IL-1β (1 ng/mL) or with TNF-α (10 ng/ml) for 6 h or 24 h. TNF-α (Figure 1(c)) and MMP-9 (Figure 1(e)) mRNA levels were markedly increased (378-fold) after 6 h or 24 h after TNF-α treatment (p < 0.001). In addition, TIMP-1 mRNA levels (Figure 1(f)) were increased (2.3-fold) after 24 h of TNF-α treatment (p < 0.001). Unexpectedly, MMP-2 mRNA levels (Figure 1(d)) were decreased after 6 h and 24 h of TNF-α treatment (p < 0.01). The levels of TAK1 protein (72 kDa), which activates p38 MAPK/JUN/NFκB signaling, were increased 24 hours after IL-1β or TNF-α treatments (Figure 1(g)). MMP-9 protein levels were increased after 24 h of IL-1β or TNF-α treatments (Figure 1(g)). TIMP1 protein levels also were increased after 24 h of IL-1β or TNF-α treatments (Figure 1(g)). Data from the migration assay indicated that neutralizing antibodies to integrin α6 (10 μg/mL) inhibited Ca9-22 cell migration that was stimulated by TNF-α (Figure 1(h)).

### TNF-α affects the formation of hemidesmosomes and desmosomes

**Figure 2.**
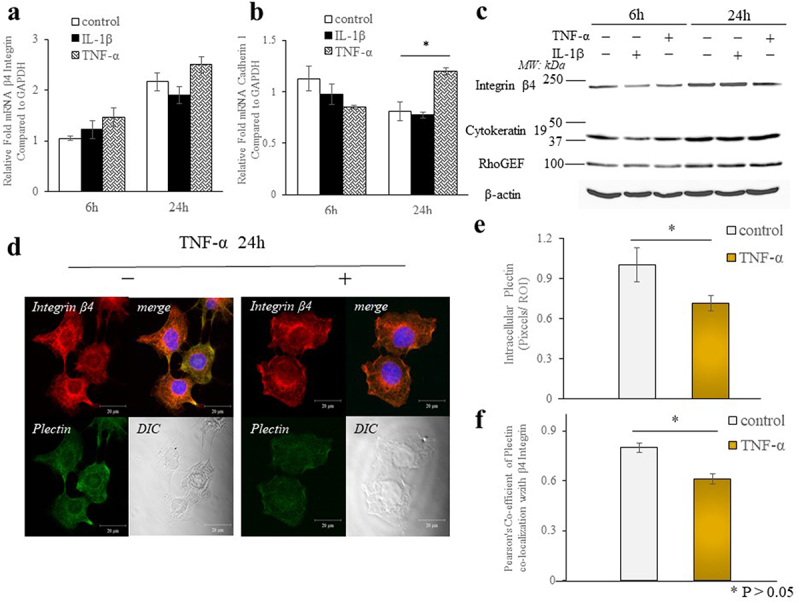
TNF-α affects the formation of hemidesmosomes. Effects of IL-1β (1 ng/ml) and TNF-α (10 ng/ml) on mRNA and protein levels of focal adhesion molecules in Ca9-22 cells. Quantification of mRNA expression of (a) β4 integrin and (b) Cadherin 1 in Ca9-22 cells that were treated IL-1β and TNF-α for 6 or 24 h before RNA extraction. Data are means SEM ± from 3 separate experiments. (c) In immunoblot analysis for β4 Integrin, cytokeratin 19 and Rho GEF protein, lysates from Ca9-22 cells. β-actin was used as a loading control. The data are representative of 3 separate experiments. * p < 0.05. (d ~ f) Collagenase treated-Ca9-22 cells were plated on fibronectin (10 μg/mL)-coated dishes and treated with TNF-α (10 ng/mL) for 24 h. They were then fixed and immunostained for plectin with permeabilization. Immunostaining for intercellular plectin and colocalization was performed by using anti-β4 integrin and anti-plectin and imaged with a Zeiss LSM 510 confocal microscope. At least 30 cells were measured for each marker and cell type. Scale bar: 20 μm. Image J Pearson coefficients were calculated to determine colocalization. * p < 0.05.

We investigated the effects of inflammatory cytokines on cell-matrix adhesions (hemidesmosome formation) and intercellular adhesions (desmosome formation) in Ca9-22 cells. Expression of the β4 integrin mRNA, a protein which plays an important role in adhesion of epithelial cells to the matrix, was not altered by the cytokines (Figure 2(a)). In contrast, the expression of the β4 integrin (202 kDa) was decreased by IL-1β (1 ng/mL) for 6 h or TNF-α (10 ng/ml) for 6 h or 24 h (Figure 2(c)). The expression of plectin, which is an important cytoskeletal protein involved in cell adhesion and that binds to the integrin β4 subunit, was studied with immunofluorescence and confocal microscopy (Figure 2(d)). Immunostained-cells were quantified on a single cell basis (pixels per region of interest) and the fluorescence intensity was normalized to specific regions of the cell. The expression of intracellular plectin was decreased (1.3-fold) by TNF-α treatment compared with controls (p < 0.05, Figure 2(e)). The colocalization of β4 integrin with plectin decreased after 24 h of TNF-α treatment (Figure 2(f)). The expression of the intermediate filament cytokeratin 19 protein (40 kDa), which is expressed in epithelial cells and binds to plectin, decreased 6 h after TNF-α stimulation, but did not change after 24 h of TNF-α stimulation (Figure 2(c)). Conversely, the expression of cadherin 1 mRNA (a protein involved in intercellular adhesion) increased 24 h after TNF-α stimulation (Figure 2(b)). We also investigated the expression of Rho GEFs, which activate Rho GTPases (Rho A, Rac1, Cdc42). These proteins are involved in the regulation of cell motility, polarity and proliferation. The expression of Rho GEFs protein levels (102 kDa) were unchanged after cytokine treatments (Figure 2(c)).

### TNF-α deregulates the composition of the basal lamina

**Figure 3.**
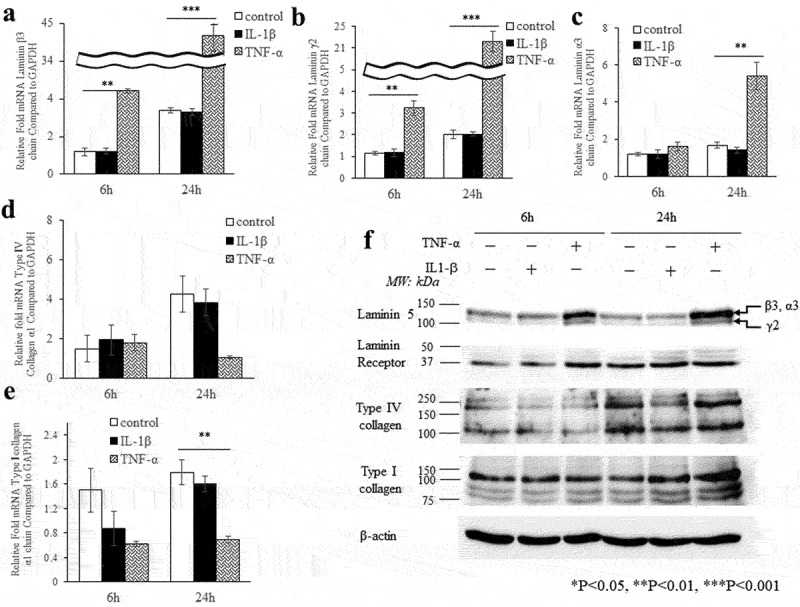
TNF-α has an effect on the basal lamina composition. Effects of IL-1β (1 ng/ml) and TNF-α (10 ng/ml) on mRNA and protein levels of basal lamina composition molecules in Ca9-22 cells. Quantification of mRNA expression of (a) laminin β3, (b) laminin γ2, (c) laminin α3, (d) type IV α1 collagen and (e) type I α1 collagen in Ca9-22 cells that were treated IL-1β and TNF-α for 6 h or 24 h before RNA extraction. Data are means SEM ± from 3 separate experiments. (f) Immunoblot analysis of laminin 5, laminin 5 receptor, type IV collagen and type I collagen from lysates prepared from Ca9-22 cells. β-actin was used as a loading control. The data are representative of 3 separate experiments. ** p < 0.01, *** p < 0.001.

We investigated the effects of IL-1β (1 ng/mL) and TNF-α (10 ng/ml) on the expression of laminin-5 and type IV collagen as these proteins are important components of the basal lamina that bind to integrins. Laminin β3 (Figure 3(a)) and Laminin γ2 (Figure 3(b)) mRNA levels were markedly increased (each 13.7-fold, 11-fold) after 6 h or 24 h of TNF-α treatment (p < 0.001). Laminin α3 mRNA levels were strongly increased (3.3-fold) after 24 h of TNF-α (p < 0.01, Figure 3(c)). The expression of laminin 5 (100 ~ 150 kDa) was increased after 6 h or 24 h of TNF-α (Figure 3(f)). Conversely, the expression of laminin receptors (30 ~ 50 kDa), which are involved in cell-matrix adhesion, increased after 6 h of TNF-α, but were only slightly increased after 24 h of IL-1β or TNF-α (Figure 3(f)). Type IV α1 collagen mRNA levels were decreased after 24 h of IL-1β or TNF-α (Figure 3(d)). The expression of type IV collagen protein (160 kDa) was decreased 24 h after IL-1β stimulation but was largely unchanged after 24 h of TNF-α (Figure 3(f)). Type I α1 collagen mRNA levels were markedly decreased after 24 h of TNF-α (p < 0.01, Figure 3(e)). Type I collagen (α1 chains; 130 kDa) was increased after 24 h of IL-1β or TNF-α (Figure 3(f)).

### TNF-α regulates the synthesis of intracellular and extracellular laminin 5 and Type IV collagen

**Figure 4.**
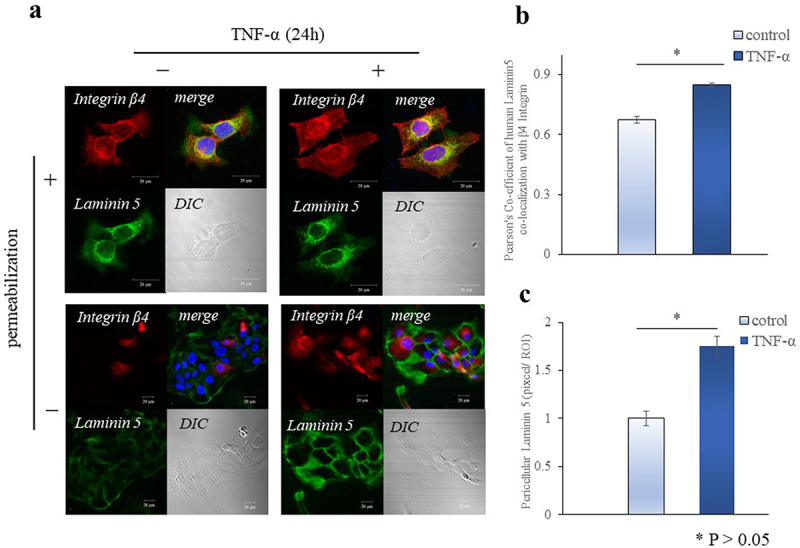
TNF-α promotes the synthesis of intra- and extracellular laminin 5. (a) Collagenase treated-Ca9-22 cells were plated on fibronectin (10 μg/mL)-coated dishes and treated with TNF-α (10 ng/mL) for 24 h. They were then fixed and immunostained for laminin 5 with permeabilization or non-permeabilization. (b and c) Immunostaining for colocalization was performed by using anti-β4 integrin and anti-laminin 5, and imaged with a Zeiss LSM 510 confocal microscope. At least 30 cells were measured for each marker and cell type. Scale bar: 20 μm. Image J Pearson coefficients were calculated to determine colocalization. * p < 0.05.

**Figure 5.**
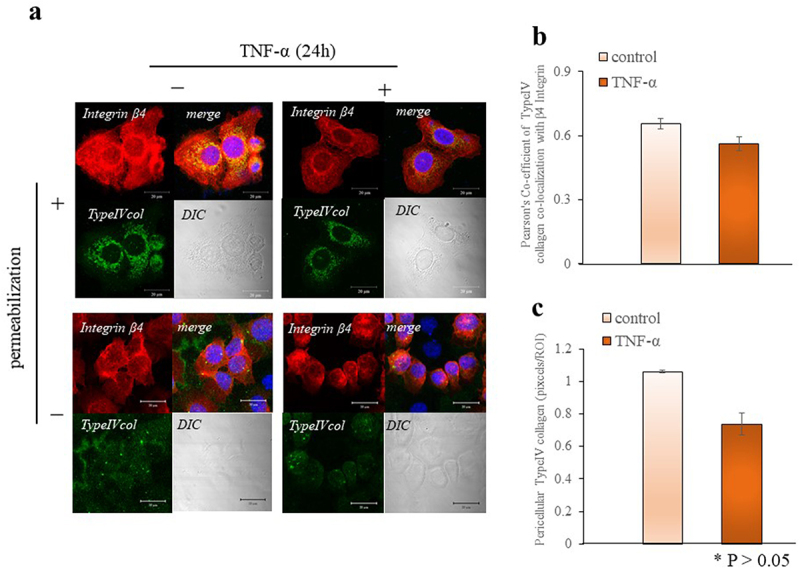
TNF-α inhibits the synthesis of intracellular and extracellular type Ⅳ collagen. (a) Collagenase treated-Ca9-22 cells were plated on fibronectin (10 μg/mL)-coated dishes and treated with TNF-α (10 ng/mL) for 24 h. They were then fixed and immunostained for type Ⅳ collagen with permeabilization or non-permeabilization. (b and c) Immunostaining for colocalization was performed by using anti-β4 integrin and anti- type Ⅳ collagen, imaged with a Zeiss LSM 510 confocal microscope. At least 30 cells were measured for each marker and cell type. Scale bar: 20 μm. Image J Pearson coefficients were calculated to determine colocalization.

We examined the co-localization of laminin 5 and Type IV collagen with integrins and the extracellular expression of laminin 5 and Type IV collagen by immunofluorescence staining. Cells were coimmunostained for laminin 5 or Type IV collagen and β4 integrin (Figure 4(a) and Figure 5(a)). The extent of colocalization was quantified by Pearson correlation analysis. Colocalization of laminin 5 and β4 integrin in Ca9-22 cells was increased (1.3-fold) after 24 h of TNF-α (p < 0.05, Figure 4(b)). Laminin 5 was immunostained in the pericellular matrix of nonpermeabilized cells, quantified on a single-cell basis by confocal microscopy (pixels per region of interest) and normalized to individual cell area. Pericellular laminin 5 increased (1.8-fold) after 24 h of TNF-α stimulation (p < 0.05, Figure 4(c)). In contrast, the co-localization of Type IV collagen and β4 integrin in Ca9-22 cells did not change after 24 h of TNF-α (Figure 5(b)), whereas pericellular Type IV collagen was slightly decreased after 24 h of TNF-α, but no significant difference was observed between two groups. (Figure 5(c)).

### TNF-α promotes the synthesis of extracellular ODAM

**Figure 6.**
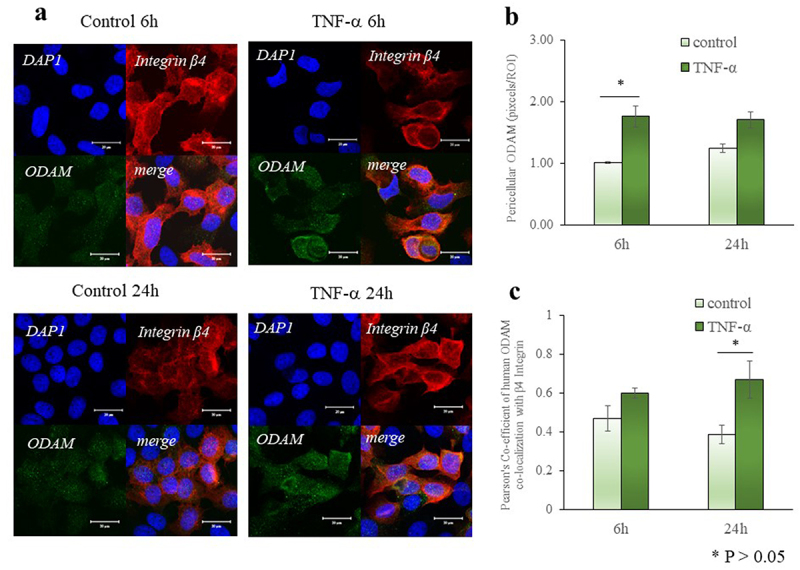
TNF-α promotes the synthesis of extracellular ODAM. (a) Collagenase treated-Ca9-22 cells were plated on fibronectin (10 μg/mL)-coated dishes and treated with TNF-α (10 ng/mL) for 24 h. They were then fixed and immunostained for ODAM without permeabilization. (b and c) Immunostaining for colocalization was performed by using anti-β4 integrin and anti- type Ⅳ collagen, and imaged with a Zeiss LSM 510 confocal microscope. At least 30 cells were measured for each marker and cell type. Scale bar: 20 μm. Image J Pearson coefficients were calculated to determine colocalization. * p < 0.05.

We investigated the extracellular expression of ameloblast-related (ODAM) by immuno-fluorescence (Figure 6(a)). Along with laminin 5 and amelotin, ODAM, which is a member of the secretory calcium-binding phosphoprotein gene family, contributes to the formation of the internal basal lamina that is located between the attached junctional epithelium and the enamel. Pericellular ODAM increased after 6 h of TNF-α treatment (p < 0.05, Figure 6(b)). Colocalization of ODAM and β4 integrin in Ca9-22 cells was increased (1.7-fold) after 6 h of TNF-α treatment (p < 0.05, Figure 6(c)).

## Discussion

The oral gingival epithelial basement membrane is comprised of classical basement membrane components including collagen IV, nidogen, laminin-5, −6, −7, −10, and −11 [13]. Periodontitis is a chronic inflammatory disease caused by bacterial, environmental, and host factors that drives progressive destruction of tooth supporting structures and in particular, the degradation of the dentogingival junction [26]. To maintain the integrity of the dentogingival junction, the internal and external basal laminae undergo continuous and rapid remodeling. This remodeling is coupled to the high turnover rates of the adjacent junctional epithelium, which is an important defense mechanism that protects the host from progressive bacterial infections [27–29]. As TNF-α is an abundant pro-inflammatory cytokine that is upregulated in periodontitis, we examined the effect of TNF-α on peri-epithelial cell remodeling. We found that the expression of laminin 5 at intra- and extracellular sites was strongly enhanced by TNF-α. Notably, there are very limited data on the effect of TNF-α on matrix remodeling by Ca9-22 cells, and there are no studies that specifically observe the extracellular expression of basal lamina proteins. In clinical studies of human patients affected by periodontitis, the laminin 5γ chain was highly expressed in the gingival crevicular fluid sampled at sites with deep periodontal pockets [30] Therefore, our data suggest that Laminin 5-mediated remodeling of the epithelial cells that comprise the dentogingival junction is enhanced by TNF-α.

We found intact ODAM in the junctional epithelium, which reflects the expression of this protein in healthy periodontal tissues and which is consistent with the earlier observation that the adhesion of the junctional epithelium to the tooth surface is mediated by fibronectin/laminin-integrin-ODAM-ARHGEF5-RhoA signaling [31]. We found increased ODAM expression in the periphery of gingival epithelial cells, which was more strongly co-localized with integrins after TNF-α stimulation. On the basis of these data we suggest that Laminin 5 and ODAM expression in the junctional epithelium may contribute to the resistance to periodontal tissue disruption that is mediated by TNF-α.

The 67 kDa laminin receptor is a non-integrin cell surface receptor that binds to extracellular matrix proteins and mediates cell adhesion to the basement membrane and provokes signal transduction after binding to the matrix [32]. Expression of the 67 kDa laminin receptor is increased in neoplastic cells and is strongly correlated with enhanced invasive and metastatic potential [33]. Our data showed increased expression of the 67 kDa laminin receptor shortly after TNF-α stimulation. These results support the involvement of laminin receptors in strengthening adhesion to ECM, as integrin β4 expression was reduced after TNF-α stimulation.

MMP-9 (also known as a Type IV collagenase, 92 kDa gelatinase, or gelatinase B) is expressed by a wide variety of cell types including epithelial cells, fibroblasts, keratinocytes, osteoblasts, dendritic cells, macrophages, granulocytes, and T-cells. MMP-9 regulates tissue remodeling by degrading ECM proteins, and it can also activate cytokines and chemokines [34,35]. We found that TNF-α strongly upregulated MMP-9 expression in gingival epithelial cells, which is consistent with earlier data showing that TNF-α promoted a dose-dependent increase of MMP-9 expression in an immortalized kidney proximal tubule epithelial cell line [36].

We studied the migration of gingival epithelial cells into a denuded area in the culture dish, which we used as a model for wound re-epithelialization (Figure 1). We found that TNF-α (24 h) treatment accelerated wound closure. We considered that the promotion of cell migration by TNF-α described here may be due to the degradation of ECM by TNF-α-induced MMP-9. Next, we investigated the distinct functions of integrin α6 during migration. Previous studies indicated that integrin α6‐neutralizing antibodies inhibited migration of human prostate cancer cells [37]. Our data showed that integrin α6 antibodies inhibited Ca9-22 cell migration after treatment with TNF-α. These data suggest that integrin α6 promotes directional migration after treatment with TNF-α. Indeed, since we also found that TNF-α reduced type IV collagen mRNA expression compared with controls, we suggest that laminin 5 and ODAM are likely to promote wound healing. Immunocompetent cells secrete pro-inflammatory cytokines like TNF-α into marginal tissues affected by periodontitis. Further, TNF-α promotes bacterial invasion of gingival tissues [38].

We found that TNF-α enhanced the expression of laminin 5 and ODAM, which are basement membrane proteins that are involved in the adhesion of the junctional epithelium to the enamel. The expression of these proteins contributes to the protection of the dentogingival junction at periodontitis sites. However, we also considered that the cells used in these studies are oral squamous epithelial cancer cells, which showed expression of cytokeratin 19 and ODAM. These proteins are specifically expressed in the junctional epithelium. TNF-α induces apoptosis via two distinct caspase-8 activation pathways of cIAP1/2 and c-FLIP [39]. Plectin is cleaved by caspase-8 at a much faster rate than other caspase substrates. Plectin is also required for the organization of the actin filament cytoskeleton in epithelial cells [40]. We found that Plectin expression by Ca9-22 cells and its co-localization with integrins were reduced by TNF-α. Although we did not observe caspase 8 expression, conceivably TNF-α-induced caspase 8 suppressed Plectin expression.

We examined the effect of IL-1β on the expression of basement membrane proteins and found a marked reduction of type IV collagen expression after 24 h of treatment with IL-1β. There were no detectable differences for the other proteins that were analyzed. Previously we found large reductions of the abundance of type IV collagen mRNA in IL-1β-treated (3 h) cells [41]. One explanation for the lack of consistency of the expression of type IV collagen and type I collagen mRNA and protein relates to the rapid degradation of extracellular type IV collagen and type I collagen by MMP-9, and by the intracellular uptake of degraded type IV collagen molecules. Since TNF-α and IL-1β inhibited collagen synthesis as expected, we suggest that inflammatory cytokines may affect the formation of external basal lamina between junctional epithelium and connective tissue and promote periodontal pocket formation.

Our major finding is that laminin 5, which is expressed by human epithelial cells, was unchanged by IL-1β treatment but was strongly increased after TNF-α treatment. We conclude that laminin 5 may play a role in the formation of the basal lamina formation by epithelial cells in periodontitis lesions in which TNF-α is strongly increased and that the observed TNF-α-driven increase of laminin 5 may indicate enhanced epithelial cell adhesion to the tooth and increased cell migration, processes that protect periodontal tissues affected by inflammatory lesions.
